# A guide to avian museomics: Insights gained from resequencing hundreds of avian study skins

**DOI:** 10.1111/1755-0998.13660

**Published:** 2022-06-23

**Authors:** Martin Irestedt, Filip Thörn, Ingo A. Müller, Knud A. Jønsson, Per G. P. Ericson, Mozes P. K. Blom

**Affiliations:** ^1^ Department of Bioinformatics and Genetics Swedish Museum of Natural History Stockholm Sweden; ^2^ Department of Zoology Stockholm University Stockholm Sweden; ^3^ Natural History Museum of Denmark University of Copenhagen Copenhagen Denmark; ^4^ Museum für Naturkunde Leibniz Institut für Evolutions‐ und Biodiversitätsforschung Berlin Germany

**Keywords:** birds, genomic libraries, museomics, natural history collections

## Abstract

Biological specimens in natural history collections constitute a massive repository of genetic information. Many specimens have been collected in areas in which they no longer exist or in areas where present‐day collecting is not possible. There are also specimens in collections representing populations or species that have gone extinct. Furthermore, species or populations may have been sampled throughout an extensive time period, which is particularly valuable for studies of genetic change through time. With the advent of high‐throughput sequencing, natural history museum resources have become accessible for genomic research. Consequently, these unique resources are increasingly being used across many fields of natural history. In this paper, we summarize our experiences of resequencing hundreds of genomes from historical avian museum specimens. We publish the protocols we have used and discuss the entire workflow from sampling and laboratory procedures, to the bioinformatic processing of historical specimen data.

## 1 INTRODUCTION

An estimated 2.5 billion specimens are housed in natural history collections worldwide (Duckworth et al., 1993) and ~10 million of these are avian study skins (Roselaar, 2003). This massive bank of information was for a long time only available for nongenetic research. However, with the advent of PCR amplification of DNA, it has become possible to obtain genetic information from very limited amounts of source material (Saiki et al., 1985). In the late 1980s, Pääbo and Wilson (1988) used the PCR technique to successfully sequence short DNA fragments from the extinct quagga *Equus quagga*. Their work pioneered the fields of museum and ancient genetics by opening the doors to using natural history collections and subfossils for genetic analyses. In the same year, Houde and Braun (1988) showed that even avian museum specimens still contain DNA molecules, and a few years later Ellegren (1991) published the first DNA sequences obtained from avian museum specimens. During the 1990s the potential of avian study skins for genetic analyses was further highlighted in several publications (Cooper, 1994; Leeton et al., 1993; Mundy et al., 1997), but avian museum samples, such as study skins, were not commonly used as a source for DNA sequencing until the late 1990s. In early studies, old avian museum samples were mainly used in phylogenetic studies, largely by generating mitochondrial DNA sequences from a few individuals (e.g., Cracraft & Feinstein, 2000; Payne et al., 2002). Soon after, studies using museum study skins began to include more samples (e.g., Fabre et al., 2012) and also included nuclear loci (Irestedt et al., 2006). Furthermore, specimens from historical populations preserved in museum collections were used for genetic comparisons with modern populations for conservation purposes (Glenn et al., 1999; Thomas et al., 1990). However, with the advent of high‐throughput sequencing the field has continued to advance (Bi et al., 2013) and has transitioned from targeted sequencing of short PCR products to shotgun‐sequencing of complete mitochondrial genomes (Guschanski et al., 2013) and whole nuclear genomes (van der Valk et al., 2019). Museomic studies have also become more common in avian research, particularly for phylogenetic and biogeographical studies, either by targeting thousands of specific loci across the genomes (McCormack et al., 2016) or by resequencing entire genomes (e.g., Ericson et al., 2019). Museomics has also proven a powerful tool for inferring population decline and inbreeding (Dussex et al., 2018) or to infer population fluctuations through time and phylogenetic relationships of extinct species (Knapp et al., 2019; Murray et al., 2017). Nevertheless, the promise of integrating museum specimens in evolutionary genomics is not a trivial exercise and both source material as well as laboratory procedures can greatly affect the yield and completeness of genomic data. DNA from historical samples is prone to the incorporation of erroneous bases due to deamination and fragmentation of the molecules (Dabney et al., 2013). As these erroneous bases can influence downstream inferences, they need to be explicitly dealt with, either before or after sequencing.

**FIGURE 1:**
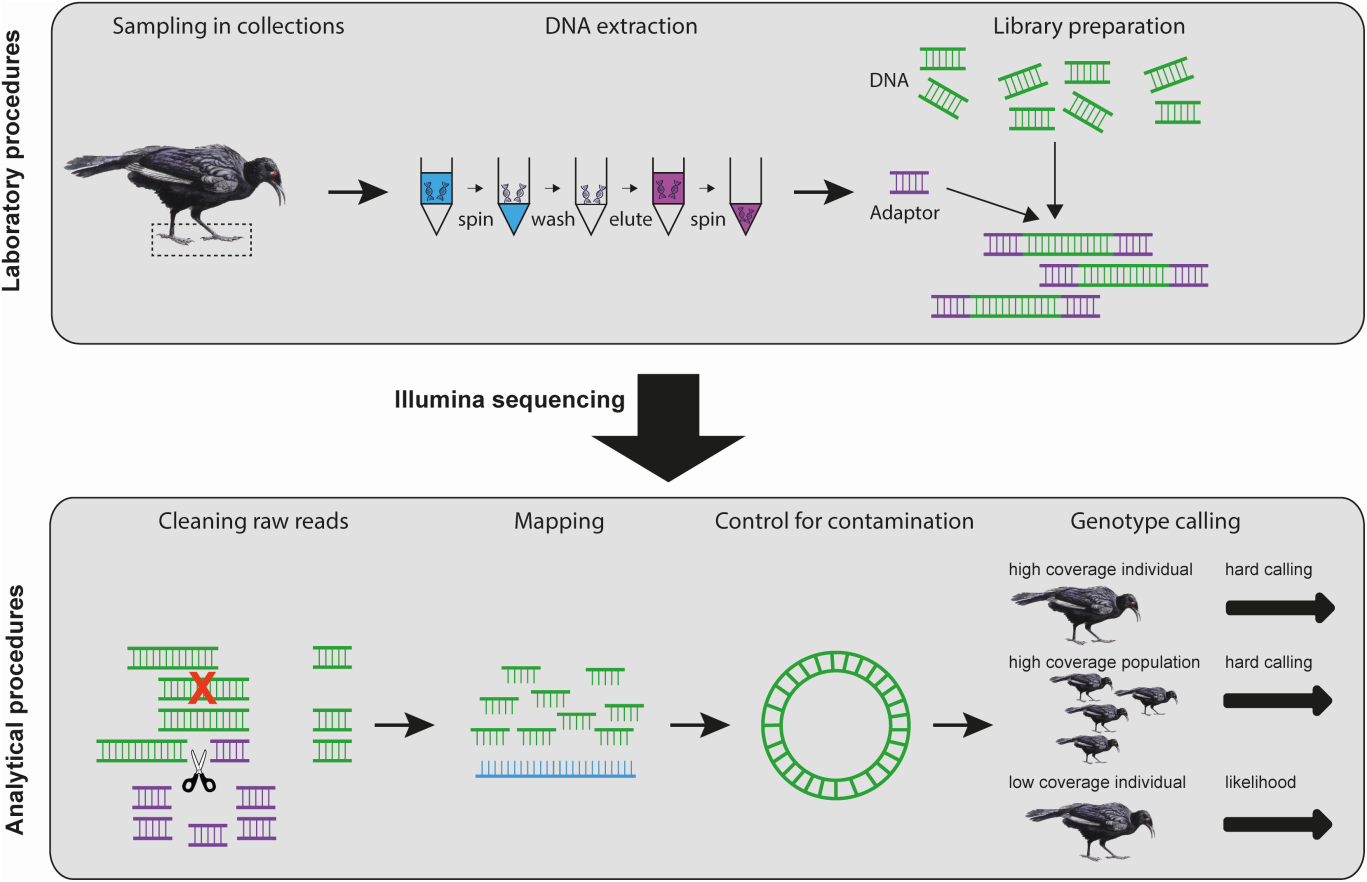
Flow chart illustrating the major steps in our museomics pipeline discussed herein. The top panel illustrates the sampling and laboratory procedures. From left to right, sampling, extraction and preparation of genome libraries for shotgun sequencing. The bottom panel illustrates the analytical procedures. From left to right, cleaning of raw reads including removal of PCR‐duplicates and adaptors, mapping against a reference genome, controlling for contamination using the mitochondrial genome, and strategies for genotype calling depending on coverage and number of individuals

In this paper, we provide an overview of our workflow and discuss the challenges associated with generating genome‐scale data sets from historical avian specimens. The paper draws on our extensive experience in obtaining genetic data from avian study skins for more than 15 years (e.g., Irestedt et al., 2006; Jonsson et al., 2007; Jonsson et al., 2012), and in particular our extensive recent work with the resequencing of entire genomes from avian study skins (see, e.g., Ericson et al., 2021; Ericson et al., 2017; Ericson et al., 2019; Ernst et al., 2022; Irestedt et al., 2019; Jonsson et al., 2019; Kennedy et al., 2022). It is not our aim to review the entire field of museomics, as there are numerous laboratory and analytical methods described in the literature both for DNA extraction (e.g., Tsai et al., 2020) and for genome library preparation from museum samples (Carøe et al., 2018; Kapp et al., 2021; Meyer & Kircher, 2010); each has its own benefits and potential drawbacks. We also do not present completely new protocols, but instead share a workflow with several modifications to existing protocols that we have used to successfully resequence the genomes from more than 700 avian study skins (to a coverage of between 6× and 20×). Given our recent experiences, the focus of this review is therefore on whole‐genome resequencing, but it is important to note that there are now also many cost‐effective methods available to subsample genomes (e.g., McCormack et al., 2016; Suchan et al., 2016) before sequencing and these are also suited for historical specimens (Billerman & Walsh, 2019). Such methods can be cost‐effective and are particularly useful when a reference genome is not readily available, or when genome‐wide data are not important. However, the focus of the present review is on whole‐genome resequencing because the availability of high‐quality avian reference genomes is expanding rapidly (see the B10K project; https://b10k.genomics.cn/), the per‐base‐pair cost of short‐read sequencing decreasing and we envisage that it may soon be cheaper to resequence entire avian genomes rather than including an additional genome reduction step. The workflow that we present and discuss includes all steps from sampling and laboratory procedures, to how we handle degradation patterns present in DNA from museum samples to minimize adverse effects on downstream analyses (Figure 1). Our hope is that this contribution will serve as a guide for anyone who intends to use museum specimens and in particular avian study skins to generate genomic data. Although the methods described herein have mainly been applied to avian study skins, the methods (sometimes with additional minor modifications) have also been applied to other vertebrates, invertebrates and plants with similar results.

## 2 SAMPLING AND LABORATORY PROCEDURES

The properties of DNA obtained from museum samples (historical or archaic) are somewhat similar to those of ancient samples (several thousand years old). However, DNA from the latter is often more fragmented, occurs in lower concentrations and has lower proportions of endogenous DNA. Thus, in the literature, it is common to distinguish between historical and ancient DNA, but confusion remains as to the interchangeable terms used for various sources of old fragmented DNA. Here we follow the definition used in several other publications (Billerman & Walsh, 2019; Raxworthy & Smith, 2021; Wandeler et al., 2007); historical DNA (hDNA) is from specimens, not originally intended as genetic resources, but archived in collections and almost always less than 250 years old. Ancient DNA (aDNA), on the other hand, is highly degraded trace DNA from naturally preserved samples that are often much older than the time span of historical collections.

### 2.1 General precautions to avoid contamination

hDNA normally occurs in rather low concentrations and has undergone various degrees of degradation. Therefore, it is important to take special precautions to minimize the risk of contamination. Particularly for aDNA, several standards have been proposed to reduce the risk of contamination and to verify the authenticity of the DNA data generated (Cooper & Poinar, 2000; Fulton & Shapiro, 2019; Green et al., 2009; Pääbo et al., 2004). Similar criteria are commonly adopted in the field of museomics (i.e., hDNA). The best‐practice protocols for working with museum samples at the Swedish Museum of Natural History include dedicated laboratory facilities for all pre‐amplification steps in which strict rules and cleaning procedures are implemented. For example, it is prohibited to enter pre‐amplification facilities if one has been in post‐amplification environments beforehand. A subsequent visit to the pre‐amplification hDNA facilities is only allowed after showering and changing of clothes. Moreover, work bench contamination is avoided by cleaning all surfaces and equipment with bleach on a regular basis. We also follow strict routines for the sampling of footpads (also known as toe pads) from avian study skins in the collections to minimize the risk of cross‐contamination between samples (e.g., place birds to be sampled on separate sheets of paper to collect falling footpad tissue and use new scalpel blades for every bird specimen sampled).

Similarly, precautions to minimize the risk of cross‐contamination are also implemented in the laboratory procedures. These include standard precautions such as always spinning down liquids such as reagents, DNA and primers before opening lids, and the use of negative controls to verify that reagents or buffers have not been contaminated. Other precautions include the preparation of aliquots from stocks of reagents/buffers to minimize the frequency of pipetting from stock solutions, and adding DNA and index‐primers at the latest stages to reduce the risk that indices, libraries or DNA extracts contaminate reagents. To minimize the risk of pipetting errors we also work with strips and use multichannel pipettes when many samples are processed in parallel. When possible, we also prepare mixes in strips, such as the dual index combination used for sequencing (see library‐preparation section below). As concentrations of hDNA may vary considerably between samples, we work separately with samples known or suspected to be particularly degraded. Although these precautions reduce the risk of contamination considerably, post‐inspection of the reads should always be conducted (see below).

### 2.2 Avian study skins as a source for DNA

The degradation of DNA in a specimen starts immediately after the individual is euthanized (for best collection practices see Blom, 2021), via both environmental, enzymatic and chemical mechanisms (Dabney et al., 2013), resulting in fragmentation and accumulation of DNA damage due to deamination of cytosines to uracils (Briggs et al., 2007; Hofreiter et al., 2001). The preservation of hDNA in museum collections can be environment‐dependent, and high temperatures and humidity (Smith et al., 2003) as well as exposure to ultraviolet (UV) light all increase the DNA degradation processes (Oroskar et al., 1993). Preservation methods such as formalin‐fixation (Stiller et al., 2016; van Beers et al., 2006) or the use of pesticides in collections (e.g., Espeland et al., 2010) can also negatively affect the preservation of hDNA. Therefore, the quality and quantity of DNA that is still present in museum samples can vary greatly between specimens and between collections. DNA quality can even vary within (e.g., soft tissue collected from different body parts) and between tissue types (e.g., bones and soft tissue), if tissues differ in the post‐preparation drying rate, or UV or pesticide exposure (Tsai et al., 2020; Zacho et al., 2021).

**FIGURE 2:**
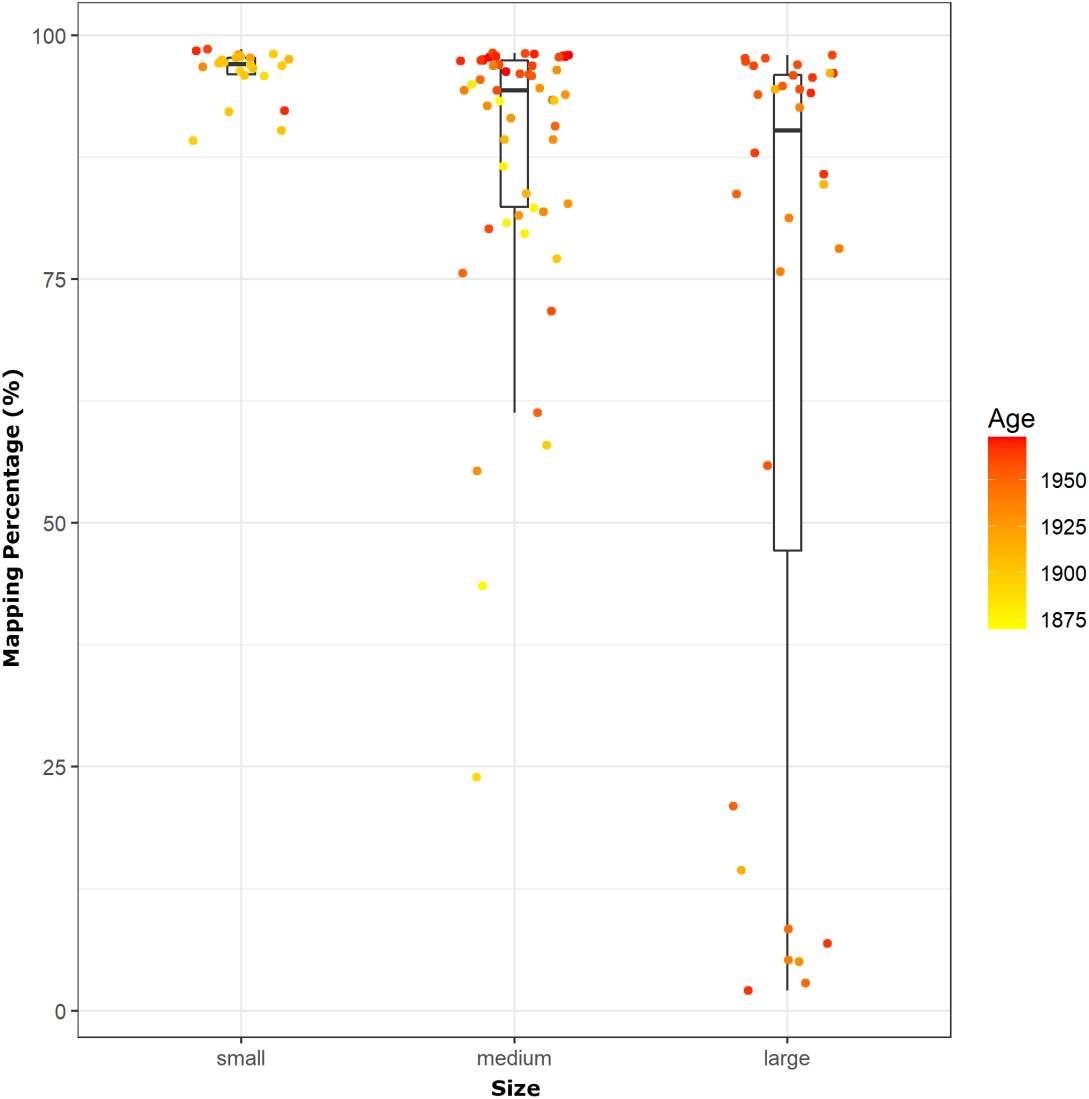
The proportion of reads from hDNA libraries from avian museum skins collected on New Guinea that map against a reference genome. The included specimens have roughly been divided into size classes: Small birds (*Pachycephala*) with a mass of ~20–40 g, medium‐sized birds (*Astrapia*, *Paradigalla* and *Drepanornis*) that weigh ~100–200 g and large birds (*Epimachus*) that weigh 150–320 g. the age of the samples is indicated by colours. Although the results show that the mapping success to some extent may be correlated with age, we find a comparably high proportion of larger birds (*Epimachus*) that map poorly, even for the youngest samples

Since the publication of Mundy et al. (1997), footpads have been the most commonly used source for hDNA from avian study skins and they have been shown to yield more DNA than other potential sources from study skins, such as skin punches and bone (Tsai et al., 2020). Our observations support this conclusion and we have now successfully sequenced the genomes of >700 birds from museum study skin footpads, including small birds such as passerines (Ericson et al., 2019) and larger birds (Cibois et al., 2019; Ericson et al., 2017, our unpublished data). However, we have observed a negative correlation between the size of the bird and the success rate in producing genome‐sequencing libraries from footpads. For example, for birds collected in New Guinea and the surrounding archipelagos (~500 individuals), we have a success rate of building genome libraries close to 100% for small passerines (>300 samples), while the success rate is about 85%–90% for larger passerines, such as certain species of birds‐of‐paradise. While we cannot make a detailed comparison in sampling strategy between specimens from the same fieldwork expedition, many of these birds have been collected during the same historical expeditions and are sourced from the same institutions (and thus stored under similar long‐term conditions). Moreover, larger birds also tend to produce libraries where the obtained sequences have a lower ratio of endogenous DNA (as indicated by a generally lower mapping ratio against the reference genomes, Figure 2). We speculate that the inverse correlation between bird size and endogenous DNA content may be caused by a slower tissue desiccation rate in larger birds, which allows for an extended period of DNA degradation and microorganismal growth following sampling. Large birds are also more likely to have been treated with arsenic, borax or citrine acid, and may have a higher fatty acid content which can enhance DNA degradation (J. Fjeldså, personal communication). This is supported by the observation that sequence data from footpad DNA of two individuals of large birds‐of‐paradise (*Epimachus*) both map poorly against the reference genome (3% and 21%, respectively), while for sequences obtained from leg tissue (that presumably has dried faster) libraries for the same individuals map very well (97% mapping success for both samples). Another observation is that recently collected samples of birds (mainly from the 1980s or later) sometimes completely fail during genome library preparation. We speculate that such specimens have been treated differently from those collected earlier (possibly due to formalin fixation of the fleshier and fatter parts of the skin, such as the feet). For very old alcohol specimens (preserved before the use of formalin for fixation) we also have a close‐to‐zero success rate, probably due to hydrolytic damage of DNA when the alcohol concentration has become too low. To conclude, our experience is that dry footpads from avian study skin samples represent an overall good source for DNA sequencing, but that other body parts such as tissue from the legs might occasionally work better, particularly for larger birds with fleshy footpads.

### 2.3 Extraction

Tsai et al. (2020) reported that they obtained a higher DNA yield from footpad samples when using phenol–chloroform than with silica spin columns, but that the average fragment length in the phenol–chloroform extracts was considerably shorter. As both fragment length and library complexity are important aspects, when resequencing genomes from hDNA, it is not clear‐cut which extraction method is to be preferred. Longer fragments generate longer read lengths, while high concentrations can reduce the number of PCR cycles needed to obtain indexed libraries with a sequenceable concentration (and thus the clonality, i.e., the ratio of multiple copies of the same fragment that needs to be discarded bioinformatically post‐sequencing). We have chosen to extract all our samples with silica spin columns partly to minimize the work with hazardous chemicals. These extracts usually have DNA concentrations (when extracted in 60 μl our samples typically have concentrations between 0.25 and 2.5 ng μl^−1^) that are high enough to avoid the need for running additional index‐PCR cycles to an extent that clonality becomes an issue (for most samples we run 7–12 cycles). We have chosen to use the QIAamp DNA Micro Kit (Qiagen), as these spin columns are optimized for eluting small quantities of DNA in low elution volumes and the manufacturer's manual is followed with a few modifications (see Appendix for our detailed extraction protocol). We have noted few problems with this extraction kit, but occasionally experienced that the use of footpads that are too large may in fact impede the extraction process rather than yield additional DNA. We hypothesize that in such instances the concentration of proteinase K and dithiothreitol (DTT) are probably too low to fully lyse the tissue.

### 2.4 Library preparation, indexing and size selection

Several protocols have been implemented for building genome libraries from aDNA and hDNA (Carøe et al., 2018; Kapp et al., 2021; Meyer & Kircher, 2010). Among these, directional ligation on single‐stranded DNA is particularly promising, as theoretically all ligated fragments can be sequenced (e.g., Kapp et al., 2021). In traditional “random” blunt‐end ligation, only half of the fragments have both the P5 and P7 adapters ligated (the other 50% will have two identical adapters, either two P5 or two P7 adapters, ligated to the ends). So far, we have only used the blunt‐end ligation protocol of Meyer and Kircher (2010), as we have found this protocol to be both cost‐effective and reliable (see Appendix for our version of this protocol). In brief, library preparation with this method consists of three steps (followed by a step of PCR indexing): blunt‐end repair, adapter ligation and adapter fill‐in. In between these steps are two size selection/cleaning steps in which either spin columns or magnetic beads can be used.

**FIGURE 3:**
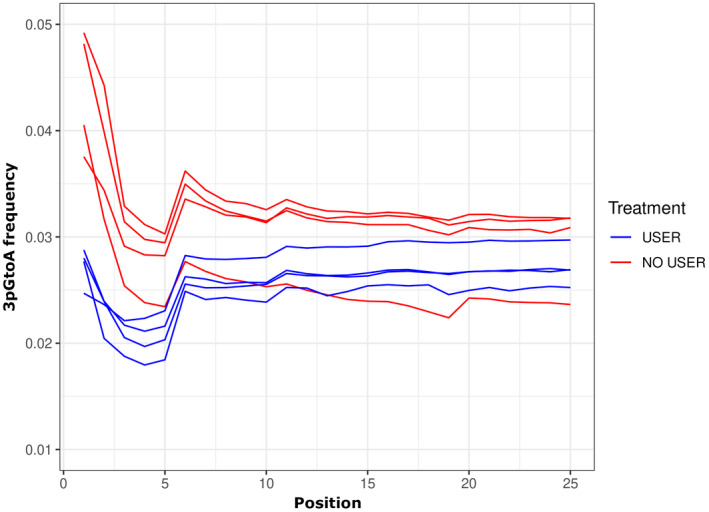
USER enzyme treatment of hDNA samples reduces damage patterns at ends of DNA fragments (especially in the outermost ~5 bp). An increased frequency of G to a substitutions at the 3′‐ends (the pattern of C to T substitutions at the 5′‐ends is similar) is evident for hDNA libraries that have not been treated with USER enzyme (red) compared to hDNA libraries that have been treated with USER enzyme (blue)

As DNA sequences from hDNA have variable error rates, primarily due to cytosine deamination forming uracil bases, it is common to enzymatically treat aDNA and hDNA samples to reduce these errors (Briggs et al., 2010). USER enzyme (New England Biolabs) uses uracil–DNA–glycosylase and endonuclease to remove uracil residues. While costly, small volumes of USER enzyme during the blunt‐end repair step (before adding T4 DNA polymerase) considerably reduces damage patterns (Figure 3) and avoids the need for hard trimming the ends on each fragment.

**FIGURE 4:**
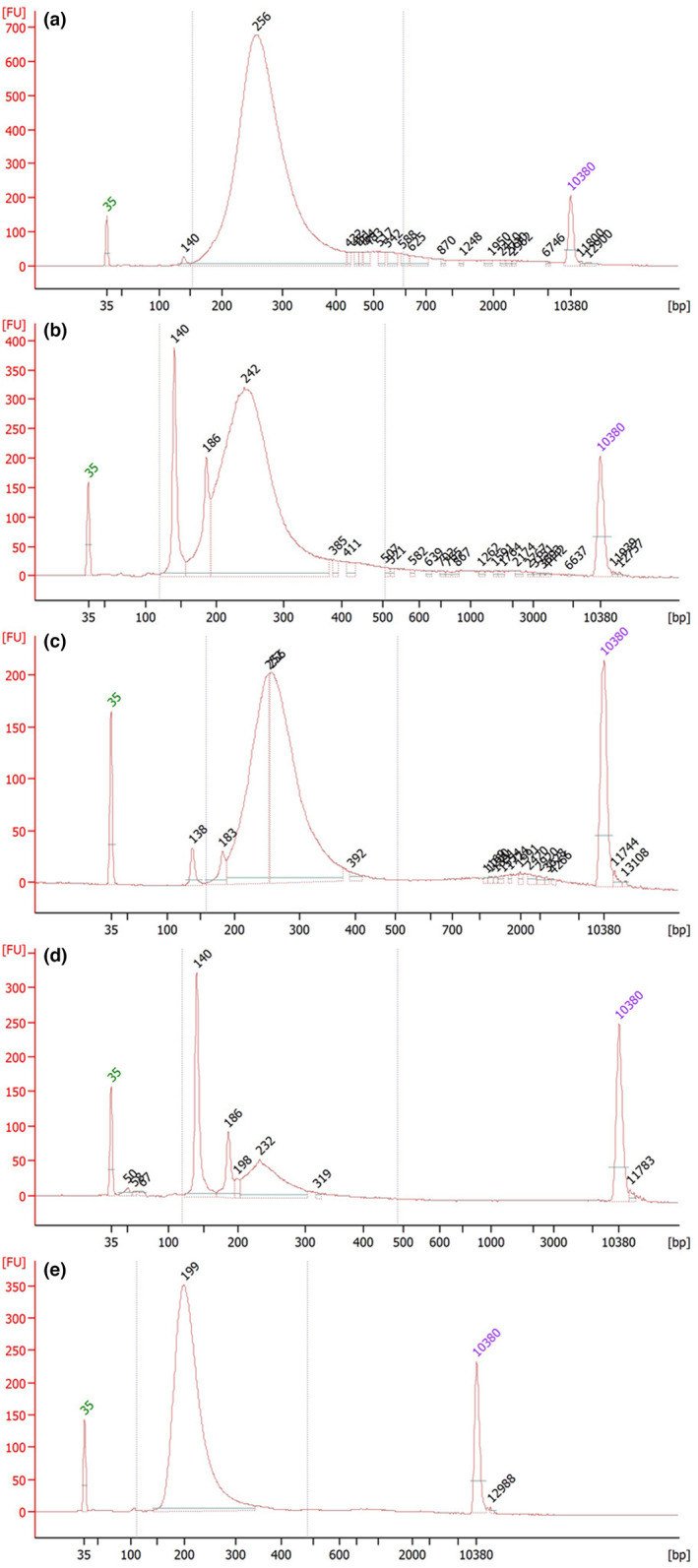
Bioanalyser output of indexed and amplified genome libraries obtained from hDNA from avian study skin samples (the *y‐*axis represents concentration and the *x*‐axis fragment lengths in bp). (a) a typical library ready for sequencing, where AMPure beads have been used for cleaning during library preparation; (b) a poor library with a lot of adapter dimers; (c) the same library as in (b), but cleaned a second time with beads in a ratio 1:1; (d) a very poor library in which the ratio between adapter dimers and library is so unfavourable that additional cleaning with AMPure beads is considered impossible; and (e) a library built using the same extract as in (d) but cleaned with MinElute columns instead of AMPure beads during the library preparation (note: The highest peak in this library is 199 bp compared to library (d) in which the equivalent peak is at 232 bp and thus considerably shorter). Peaks at 35 and 10,380 bp are the lower and upper size markers, respectively. Peaks at 140 bp are fragments consisting of ligated P5 and P7 adapters and peaks at 186 bp consists of ligated P5 and P7 adapters that have become indexed during the amplification

For the adapter ligation step, we have largely followed the recommendations for low‐concentration DNA samples by Meyer and Kircher (2010) but have reduced the amount of adapter mix (see Appendix for our library preparation protocol). We have not tested the impact of diluting the adapter mix further or made any other changes to this protocol, as the majority of our samples have worked well with the present protocol (see Figure 4a for normal library after index‐PCR and final bead cleaning).

We have observed the largest difference in performance during the two size‐selection/cleaning steps when comparing beads with MinElute spin columns. From a subset of bird‐of‐paradise footpads we have observed an average read‐length of 88 bp from libraries cleaned with spin columns (21 individuals), compared to 127 bp from libraries cleaned with beads (15 individuals). For five samples prepared using both cleaning methods, we obtained an average read length of 87 bp with spin columns and 130 bp with beads. Although we use the proportion of beads (1.8×) that should retain the same distribution of DNA fragments, we seemingly lose ~50% of the read‐length when using spin columns (even though MinElute spin columns should retain fragments between 70 bp and 4 kb). Despite apparent advantages of beads over spin columns, they are less efficient in removing free adapters in poor samples. Thus, we occasionally get high adapter peaks after the final cleaning of the indexed libraries (Figure 4b). As adapter dimers interfere with the sequencing yield (a large proportion of sequenced reads will be adapter dimers), libraries with excess adapter dimers are not optimal for sequencing. In most cases these adapter peaks can be reduced by additional steps of bead cleaning with lower proportions between libraries and beads (Figure 4c), but for very poor samples the proportion of adapter dimers can be so high that it is virtually impossible to remove enough dimers without losing the actual library (Figure 4d). In such cases, the more efficient removal of adapters with MinElute spin column cleaning during library preparation is more suitable, but comes at the expense of reducing fragment length (Figure 4e). Consequently, and in line with the recommendations by Meyer and Kircher (2010), our experience is that bead cleaning during library preparation is preferred for most hDNA extracts from avian footpads (as one obtains considerably longer reads). Further dilution of the adapter mix or a reduction of the proportion of beads used during the second cleaning step might have similar effects.

As ligated fragments are randomly amplified in the index‐PCR step, a higher number of PCR cycles will increase the clonality (that certain fragments are overrepresented in the final indexed library). To circumvent this problem, we reduce the number of cycles as much as possible (we normally run 7–12 cycles) and run multiple independent PCRs (normally four) per sample. To control for index hopping (when an index sequence for one sample is incorrectly assigned to a different sample in a pool), we also use dual indexing where both the P5 and the P7 adapters are indexed with unique barcodes (van der Valk et al., 2020). Using dual indexing allows for independent post‐cleaning of raw reads from uniquely indexed libraries before all reads per individual are pooled (see below).

Before pooling all samples according to the number of reads that are desired for sequencing, the four indexed PCR products are pooled together for each individual and then cleaned using beads. Our experience is that using a proportion of 1:1 between the pooled indexed PCR products and beads normally reduces adapter dimers sufficiently.

### 2.5 Sequencing strategies

**FIGURE 5:**
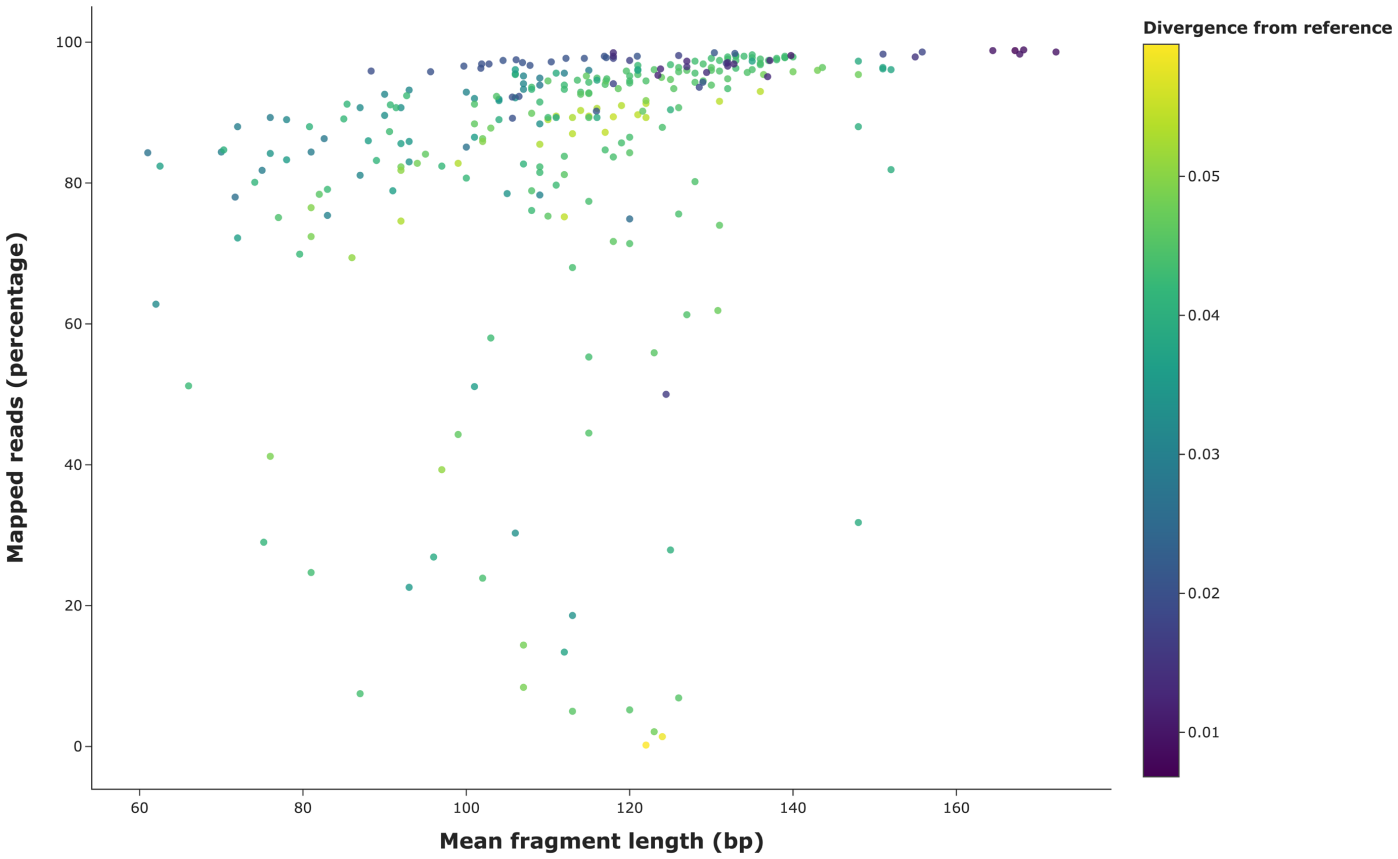
Relationship between fragment length of orphan reads, mapping efficiency and divergence from reference, based on shot‐gun sequencing of close to 300 avian museum skins. The short DNA fragments usually recovered from historical specimens have a substantial overlap between forward and reverse reads when conducting paired‐end sequencing. Following merging, the resulting orphan reads are mapped with bwa‐mem and mapping efficiency, the percentage of reads with a unique hit out of all reads, tends to increase with increasing fragment lengths. Moreover with increasing fragment lengths, the divergence between the focal individual and reference genome has less of an effect on mapping efficiency (within the divergence range of 0.03–0.06)

Sequencing strategy, the number of individuals and sequencing coverage per individual, is a trade‐off between project objectives, the corresponding downstream analyses and the amount of available funds. One confounding factor may be that the proportion of endogenous DNA may be low. Thus, it is common practice to screen such samples through an initial round of shallow Illumina resequencing to assess the proportion of endogenous DNA. By doing so one can exclude poor samples and continue deep resequencing only using the best samples. However, our experience is that DNA extracts from avian footpads generally have high proportions of endogenous DNA as evident from the high percentage of reads that map against the reference genomes (in general between 80% and 95%, see Figure 5 and Figure S1). We thus do not conduct prescreening on a regular basis.

However, screening can be a useful tool to assess more precisely how much sequence data are needed to achieve a specific coverage for an important sample or when sequencing a batch of samples from a new collection/different tissue type. As shown herein, large birds with fleshy footpads may be candidates for prescreening. Screening could also be sensible if one plans to use many samples from collections that the researcher is not familiar with, as the quality of DNA may vary considerably between collections. The libraries themselves can to some extent also provide information as to how well a particular sample will sequence. As samples that produce poor libraries have in general low DNA concentrations, it is likely that background DNA has built up higher proportions of the total DNA. Poor DNA quality also indicates that the degradation in the sample has been particularly severe, probably involving putrefaction and growth of microorganisms.

The S4 flow‐cell on the Illumina Novaseq platform is currently per basepair yield the cheapest option for genome sequencing and is our preferred set‐up. However, due to the high sequencing output of S4 flow‐cells (600–800 Gb), initial investments are needed so that sufficient samples can be multiplexed in a single sequencing run to make this cost‐efficient. Other Illumina platforms may be more suitable for smaller projects; for example, if one is aiming for a few genomes at relatively low coverage or only the mitochondrial genomes (which normally have tens to hundreds times higher coverage than nuclear genomes). As a rule of thumb, we pool 24 individuals (using four dual‐indexed libraries per individual) when we aim for 7–8× coverage (or 12 samples when aiming for ~15× coverage) when sequencing on one lane of the S4 flow cell on the Illumina NovaSeq platform. With current prices, the sequencing cost for a 7–8× coverage genome from a footpad sample is then around 180 EUR.

## 3 ANALYTICAL PROCEDURES

DNA damage in historical specimens can be characterized as fragmentation and deamination patterns towards the ends of the DNA fragments. Bioinformatic workflows that process sequencing libraries from historical samples should be specifically tailored to characterize the extent of damage and take the resulting biases into account during downstream analyses. Nonetheless, it is important to note that hDNA samples differ substantially from aDNA samples, and that appropriate pipelines therefore also vary. For example, the ratio of endogenous DNA recovered from avian footpads regularly exceeds 90% and the mean of the size distribution of DNA fragments ranges from 100 to 120 bp. The bioinformatic approach that we commonly follow is therefore more similar to pipelines suited for fresh tissue samples, but we have incorporated a number of adjustments in our workflow to account for fragmentation and deamination. Finally, the importance of sequencing coverage should not be underestimated, particularly for historical specimens for which accurate variant calling can be difficult due to the additional “noise” in hDNA (deamination, erroneous mapping of short‐reads, etc.). Bioinformatic workflows can only optimize results given the data available and the accuracy of downstream inferences therefore scales with the quality of the data produced.

### 3.1 Cleaning of raw reads

Nextflow is a scalable and reproducible scientific workflow programming model that can easily be implemented in local environments or expanded to take advantage of high‐performance computing cluster environments (Di Tommaso et al., 2017). We have developed a custom pipeline to process hDNA sequencing libraries and recently converted the entire cleaning workflow to a Nextflow pipeline (nf‐polish; https://github.com/MozesBlom/nf‐polish). nf‐polish processes each sequencing library separately, rather than by individual, and we only merge libraries once they have been mapped to a reference. Prior to any library modification, nf‐polish quantifies the degree of (adapter) contamination and sequencing quality using fastqc (version 0.11; Andrews, 2010). Adapter “read‐through” is commonplace due to the short size of the DNA fragments and the sequencing set‐up frequently used by sequencing facilities (e.g., 150‐bp paired‐end sequencing). Following an initial round of quality control, nf‐polish uses superdeduper (Petersen et al., 2015, Now part of htsstream, version 1.3) to remove PCR duplicates. Deduplication can be done by comparing each read in a library to each other (e.g., superdeduper; computationally highly intensive) or by comparing the start coordinates of read‐pairs following mapping (e.g. picard‐dedup; computationally less demanding). However, for hDNA, coordinate‐based deduplication probably leads to a high proportion of false positives since only the start position of each read(−pair) is used to identify duplicates. Paired‐end reads have two starting coordinates (the first position in the 5′ direction of both the forward and reverse read) but cleaned hDNA libraries have a high proportion of merged reads (leading to an effectively single‐end data set with a single starting coordinate at the 5′ position). With duplicates largely removed, nf‐polish then trims away frequently used Illumina sequencing adapters (trimmomatic version 0.39; [Bolger et al., 2014]) and merges read‐pairs with a substantial overlap between forward and reverse read (pear version 0.9; Zhang et al., 2014). Merging (and deduplication) is done because each mapped read should correspond to a unique DNA molecule to avoid over‐inflation of coverage or a nonbiological skew in coverage for one haplotype. Moreover, merging improves the quality score of that fragment and is therefore done prior to quality trimming (trimmomatic). The final cleaning step includes the removal of low‐complexity reads (>50% of one nucleotide type) since these often stem from highly repetitive regions, are difficult to reconstruct and are prone to mapping error. Following each processing step, nf‐polish calculates processing statistics with seqkit (version 0.16; Shen et al., 2016) and finally visualizes the modifications throughout the workflow with a custom set of plotting functions.

### 3.2 Control contamination using mitochondrial genomes

Cross‐contamination between libraries is a major concern in any genomic project. Incorporation of heterospecific DNA can complicate variant calling, lead to an excess in heterozygosity and have substantial consequences for various analyses. This is particularly relevant for aDNA and hDNA projects where the potential ratio of contaminant to endogenous DNA is usually higher and may therefore have a stronger impact on variant calling. The time invested in safe laboratory practices will represent only a fraction of the potential time lost trying to rescue a contaminated sample. However, even when applying the best practices, cross‐contamination can still occur, be it during tissue sampling, DNA extraction, library preparation or sequencing. A bioinformatic evaluation step for cross‐contamination is therefore a worthwhile exercise.

Identifying possible instances of contamination is challenging, particularly when the coverage and/or complexity of a library is low. The mitochondrial genome is a particularly useful molecule for contamination investigation. The copy number of the mitogenome is several fold higher than the nuclear genome in each cell, and sequencing coverage for the mitogenome is thus much higher in many shotgun‐sequencing projects. Moreover, as mitochondrial genomes are haploid, one can suspect contamination if heterozygote sites are called when assembling mitochondrial genomes. While it is also possible that nuclear mitochondrial loci (NUMTs) or heteroplasmy may result in some heterozygous sites, these should generally be rare and highly localized in any mitogenome. DNA damage may also lead to heterozygous calls but can be controlled for due to the relatively higher sequencing coverage of mitogenomes compared to nuclear loci.

In our workflow, we either map resequence data directly to a close mitogenome reference (if available) or use an iterative baiting and mapping approach for pseudo‐de novo assembly (e.g., mitobim version 1.3; Hahn et al., 2013). Once we have retrieved a (partial) mitogenome for a given individual, we then do a second round of mapping and variant calling using the new reference and evaluate how many reads in a library support an alternative genotype call. Since the mitogenome is a haploid molecule and the mapped libraries are from the same individual, no variants should be recovered after filtering for randomly distributed variant calls (either deamination or sequencing errors). Consistent support for heterozygous or alternative genotypes is indicative of cross‐contamination, assembly errors or heteroplasmy and should be further investigated.

### 3.3 Genotype (likelihood) calling and filtering

Variant calling and filtering are arguably among the most challenging workflow aspects in many avian genomics projects. For fresh tissue samples, variant calling and filtering is mostly conducted to exclude sequencing errors and to identify true variants from background noise. However, for hDNA, this is further complicated by DNA substitutions introduced post‐mortem. Such DNA changes are therefore not a technical artefact, but still biologically of little interest and should be excluded from downstream analyses. The challenge of variant calling and filtering is often also exacerbated by a lack of coverage in hDNA sequencing projects. Merging overlapping read‐pairs, deduplication due to low‐input amounts and mapping issues due to short fragments all reduce the amount of coverage that can be obtained from a standard sequencing run. Variant calling and filtering should therefore be carefully weighed given the available data set (coverage, DNA damage, etc.) and the suitability of the downstream analyses evaluated. For example, while phylogenetic or systematic inferences may be less affected by variant call uncertainty, demographic analyses that model changes in allele frequencies may be substantially changed. Here we briefly outline three different pathways for variant calling that are not necessarily mutually exclusive. We frequently employ multiple approaches to assess the robustness of our eventual findings.

#### 3.3.1 Hard‐calling by individual

If coverage permits (>~15×), variant calling can be performed for each individual using variant call software such as gatk (Van der Auwera & O'Connor, 2020), bcftools (Li et al., 2009) or freebayes (Garrison & Marth, 2012). However, in contrast to projects focusing on model organisms, hDNA projects often focus on organismal groups for which no training data are available and variant calling is done entirely unsupervised. It is therefore paramount to carefully weigh the resulting variant call set with post hoc filtering. When filtering by individual, several filtering metrics should almost always be employed such as coverage, allele‐frequency observations and quality (e.g. using vcftools; Danecek et al., 2011).

#### 3.3.2 Hard‐calling by population

When multiple samples are sequenced per population, variant callers such as freebayes and gatk allow a neutral model of allele diffusion to be used to improve genotyping based on a population prior. In other words, the probability of a given genotype is determined depending on the frequency of the same allele(s) in the rest of the population. Moreover, the resulting variant call set can then also be filtered by individual and population simultaneously. For example, a questionable variant call can be excluded if a certain individual is the only individual with that specific allele or when a site has more than two alleles. Similarly, a consistent multifold increase in coverage for a given locus across many individuals may be a signature of a paralogous gene or repetitive region and would be worthwhile to exclude from downstream analyses.

#### 3.3.3 Genotype likelihoods

Rather than hard‐calling genotypes, that is explicitly assigning a genotype at a given site for each individual, an alternative approach is to estimate genotype likelihoods or probabilities. Genotype likelihoods aim to take the uncertainty surrounding a certain genotype call into account and use this information in downstream inference approaches such as population structure estimation or demographic inference. This is particularly relevant for aDNA (or some hDNA) studies where coverage per individual may be low. angsd (Korneliussen et al., 2014) is a software tool that is of particular interest since it can both estimate genotype likelihoods (using several models), and filter the resulting calls while it simultaneously provides a framework for frequently used analyses in population genetics.

## 4 CONCLUSIONS

With the increasing ease and cost efficiency of integrating high‐throughput sequencing, natural history museum resources are now accessible for genomic research. In this guide, we present a workflow for integrating museum specimens at a large scale and demonstrate how intrinsic properties of hDNA, such as damage patterns and increased risk of contamination, can be dealt with in the laboratory and bioinformatically for an optimal output. The purpose of this guide is to facilitate genomic research on natural history collections, as these collections hold an enormous potential to address biological questions.

## AUTHOR CONTRIBUTIONS

M.I. and M.P.K.B. conceived the study. All authors contributed to build the data set. M.I., F.T. and K.A.J. performed bioinformatics and made the Figures. M.I. and M.P.K.B. led the writing, and all authors contributed to the discussion of the results and the writing of the manuscript.

## CONFLICT OF INTEREST

The authors declare no conflicts of interest.

## BENEFIT‐SHARING STATEMENT

Benefits from this research accrue from the sharing of our laboratory protocols and experience to conduct genome sequencing from avian hDNA samples. Data and results from all our studies that this paper are built on are available on public databases as described above.

## Supporting information


Figure S1

Appendix

## Data Availability

This study draws mainly on long‐term laboratory experience of laboratory procedures on hDNA. The data used to produce some of the figures are from our own already published studies (see original publications to get access to raw data) and some forthcoming publications.
