# Supplementary material for: A guide to avian museomics: Insights gained from resequencing hundreds of avian study skins
Source: Mol Ecol Resour. 2022 Jun 23;22(7):2672–84. doi: 10.1111/1755-0998.13660 (PMC9542604; doi:10.1111/1755-0998.13660)

FIGURE S1. Relationship between fragment length of orphan reads and mapping efficiency, based on shot-gun sequencing of close to 300 avian museum skins. The figure is identical to Figure 5 except for that color-codes for divergence time are replaced by color-codes for taxonomic groupings. In most cases, the codes represents a genera or a family, while APDE includes the genera *Astrapia*, *Epimachus*, *Drepanornis* and *Paradigalla*, BOP all other birds-of-paradise genera and Vultures the genus *Coragyps*.


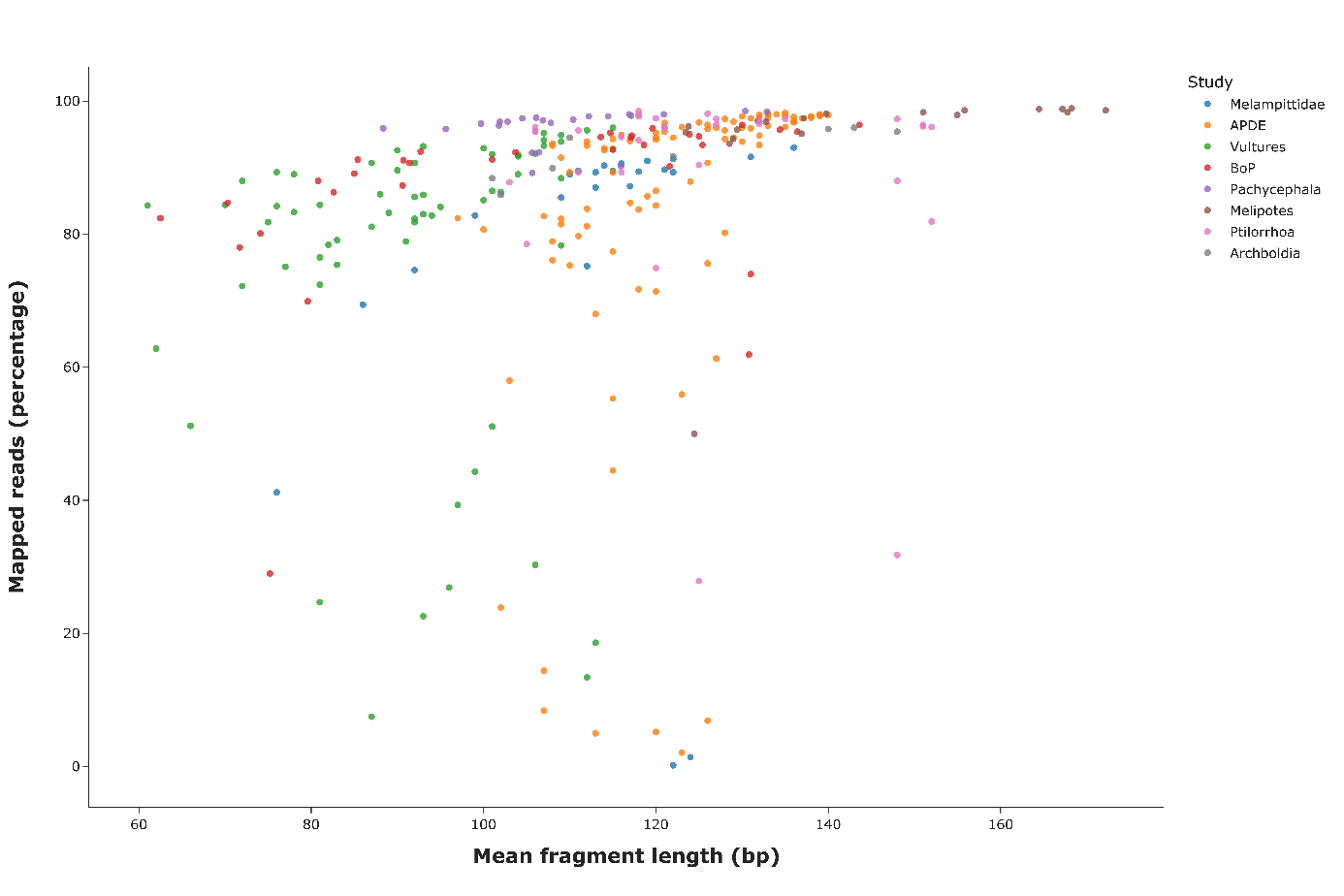

Supplement: Supplementary file 1 — Figure S1 [file MEN-22-2672-s001.docx]
