# Supplementary material for: A guide to avian museomics: Insights gained from resequencing hundreds of avian study skins
Source: Mol Ecol Resour. 2022 Jun 23;22(7):2672–84. doi: 10.1111/1755-0998.13660 (PMC9542604; doi:10.1111/1755-0998.13660)
Supplement: Supplementary file 2 — Appendix [file MEN-22-2672-s002.docx]

# DNA extraction protocol for avian museum foot pad samples using QIAamp DNA Micro Kit

*[The protocol is a modification of the manufactures tissue protocol]*

1. Put samples in ddH_2_O (ca 100-200 μl) to soften them up for a while (30-60 minutes).

2. Cut up of tissue with a scalpel knife (~5 - 20 mm^3^), and place in a 1.5 ml microcentrifuge tube.

3. Immediately add 180 μl Buffer ATL.

4. Add 20 μl Proteinase K and 20 μl 1M DTT. Mix tby pulse-vortexing (~10 s) and incubate overnight (~16 h) in a thermomixer at 56 °C and 200 - 300 rpm.

5. Add additional 5-7 μl Proteinase K and incubate another 30-60 minutes depending on how well the sample lysed overnight.

6. Add 200 μl Buffer AL, close the lid, and mix by pulse-vortexing. Incubate at 72 °C for 10 minutes.

7. Add 200 μl 96-100% EtOH, close the lid, and mix thoroughly by pulse-vortexing for 15 s. Incubate for 5 min at room temperature (RT).

8. Briefly centrifuge the 1.5 ml tube to remove drops from inside the lid.

9. Carefully transfer the entire lysate from step 8 to the QIAamp MinElute column (in a 2 ml collection tube). Close the lid, and centrifuge at 6000 x g for 1 min. Place the QIAamp MinElute column in a clean 2 ml collection tube, and safely discard the collection tube containing the flow-through.

10. Carefully open the QIAamp MinElute column and add 500 μl Buffer AW1. Close the lid, and centrifuge at 6000 x g for 1 min. Place the QIAamp MinElute column in a clean 2 ml collection tube, and safely discard the collection tube containing the flow-through.

11. Carefully open the QIAamp MinElute column and add 500 μl Buffer AW2. Close the lid, and centrifuge at 6000 x g for 1 min. Place the QIAamp MinElute column in a clean 2 ml collection tube, and safely discard the collection tube containing the flow-through.

12. Centrifuge at full speed (20 000 x g) for 3 min to dry the membrane completely.

13. Place the QIAamp MinElute column in a clean 1.5 ml microcentrifuge tube and discard the collection tube containing the flow-through. Carefully open the lid of the QIAamp MinElute column and apply e.g. 30 - 60 μl Buffer AE to the centre of the membrane (20 μl is needed for one library preparation).

14. Close the lid and incubate at RT for 5 min. Centrifuge at full speed (20 000 x g) for 1 min, the 1.5 ml microcentrifuge tube now contains the extracted DNA, discard the QIAamp MinElute column.

# Library Preparation of degraded DNA for Illumina Sequencing using bead cleaning

*[This protocol is based on Meyer & Kircher 2010] Note: The final results depend on the quality of the DNA-extract. A higher DNA concentration may reduce the number of PCR-cycles needed during the Library PCR. One solution to this is to concentrate your DNA-extract by eluting in small volumes or concentrate it with AMPure beads before proceeding with the library preparation. By adjusting the Volume of beads one may also be able to shift the average fragment size towards longer (or shorter) fragments.*

## Buffers that should be pre-prepared

### *Adapter mix stock solution (100 µl for 200 reactions) (after Meyer & Kircher)*

First prepare an Oligo hybridization buffer (200 reactions):

500 mM NaCl

10 mM Tris-Cl pH 8 (Trizma from Sigma)

1 mM EDTA pH 8

Make hybridization mix for adapter P5 and adapter P7 in PCR tubes

Hybridization mix for adapter P5 (200 µM):

40 µl of 500 µM IS1_adapter_P5.F

40 µl of 500 µM IS3_adapter_P5+P7.R

10 µl of 10X Oligo hybridization buffer

10 µl ddH_2_O

Hybridization mix adapter P7 (200 µM):

40 µl of 500 µM IS2_adapter_P7.F

40 µl of 500 µM IS3_adapter_P5+P7.R

10 µl of 10X Oligo hybridization buffer

10 µl ddH_2_O

Mix and incubate the reactions for 10 s at 95 ^o^C followed by a steady decrease from 95 ^o^C to 12 ^o^C at a rate of 0.1 ^o^C/s.

Combine both reactions to get a ready-to-use mix (with 100 µM of each adapter) and store the stock in the freezer.

### *Bead buffer*

PEG-8000 powder (Promega V3011) 9 g (8.30 ml)

5 M NaCl 10 ml

1 M Tris-HCl, pH 8.0 500 µl

0.5 M EDTA, pH 8.0 100 µl

ddH_2_O 30.1 ml

Tween-20 (100%) 27.5 µl

Add 9 g PEG-8000 to a 50 ml Falcon tube. Add NaCl, Tris-HCl, EDTA, and ddH_2_O to the powder. Shake the Falcon tube until all PEG has dissolved. Add Tween-20 and mix properly. The bead buffer should be kept in dark and in a fridge.

## Library preparation step 1 - Blunt End Repair with USER (40 µl final volume/reaction)

*Note: Always prepare mix for one extra sample to include as negative control*

*Mix carefully by flicking the tube with a finger, avoid vortexing after addition of enzymes!*

1. Prepare master mix:

| Blunt-End Repair master mix (with USER): | | |  |
| --- | --- | --- | --- |
| Reagent | Volume/sample [µl] | Final concentration |  |
| Tango Buffer (10X) | 4 | 1X |  |
| dNTPs (25 mM) | 0.16 | 100 µM |  |
| ATP (100 mM) | 0.4 | 1 mM |  |
| T4 PNK (10 U/µl) | 2 | 0.5 U/µl |  |
| USER | 3 |  |  |
| ddH_2_O | 9.64 |  |  |
| Total master mix: | 19.2 |  |  |

2. Using a single- or multichannel pipette, add 19.2 µl of master mix to 20 µl of DNA extract.

3. Mix thoroughly and incubate in a thermal cycler for 3 hours at 37 °C.

4. Add 0.8 µl of T4 DNA Polymerase (5 U/µl, final concentration 0.1 U/µl) per sample.

5. Mix and incubate in a thermal cycler for 15 min at 25°C, followed by 5 min at 12°C.

6. Place plate on ice or immediately proceed to the next step.

### Purification with AMPure beads

7. Add 72 µl AMPure beads (1.8X) to the 40 µl of repaired DNA.

8. Vortex, pulse spin and incubate for 5 min.

9. Place the tubes on a magnetic rack/plate.

10. When all beads are attached to the wall remove the supernatant (DNA is attached to the beads).

11. Clean the beads by adding 200 µl fresh 70% ethanol.

12. Vortex, pulse spin and place the tubes on a magnetic rack/plate.

13. When all beads are attached to the wall remove the ethanol.

14. Repeat the ethanol cleaning one more time.

15. After the final washing keep the tubes open (about 5 min) to let the remains of ethanol evaporate.

16. When the bead-pellet look like mud add 20 µl EB.

17. Vortex, pulse spin and incubate for 5 min.

Note: The beads should be kept in the solution when continuing with the adapter ligation

### *Alternative purification with MinElute spin columns for low concentration and poor samples*

*a. Apply 200 µL of PB Buffer to 40 µL of the repaired DNA. Mix, and then add to MinElute column.*

*b. Spin 13 000 rpm for 1 min*

*c. Discard waste and change to new collection tube. Discard PB Buffer separately!*

*d. Add 700 µL of PE Buffer to column. Spin 13 000 rpm for 1 min. Discard waste and change to new collection tube. Repeat this PE wash one more time (2 washes in total).*

*e. Spin 13 000 rpm for 1 min to dry the column*

*f. Change to new 1.5 mL tube*

*g. Leave column with open lid for 5 min to dry out column*

*h. Add 22 µL of EB Buffer to column*

*i. Incubate for 5 min at 37°C*

*j. Elute DNA by spinning it down for 1 min at 13 000 rpm*

## Library preparation step 2 - Adapter Ligation (40µl final Volume/reaction)

Prepare a master mix (20 µl/reaction) for the required number of ligation reactions as shown below. Keep enzymes on ice. If white precipitate is present in the 10X DNA ligase buffer after thawing, warm the buffer to 37 °C and vortex until the precipitate has dissolved. Since PEG is highly viscous, vortex the master mix before adding T4 DNA ligase and mix gently thereafter. Finally mix 20 µl of the master and 20 µl of the DNA per reaction.

| Reagent | Volume/sample [µl] |  |  |
| --- | --- | --- | --- |
| ddH_2_O | 10 |  |  |
| T4 DNA ligase buffer (10X) | 4 |  |  |
| PEG-4000 (50%) | 4 |  |  |
| Adapter mix (1µl of a 1:10 dilution in TE of the stock = 10pmol in final Volume) | 1 |  |  |
| T4 DNA ligase (5 U/µl) | 1 |  |  |
| Total master mix: | 20 µl |  |  |

18. Mix and incubate for 30 min at 22°C

### Purification with AMPure beads (and removal of beads)

19. Add 72 µl of the pre-prepared Bead buffer (1.8X) to the 40 µl ligated DNA (that already contains beads).

20. Vortex, pulse spin and incubate for 5 min.

21. Place the tubes on a magnetic rack/plate.

22. When all beads are attached to the wall remove supernatant.

23. Clean the beads by adding 200 µl fresh 70% ethanol.

24. Vortex, pulse spin and place the tubes on a magnetic rack/plate.

25. When all beads are attached to the wall remove the ethanol.

26. Repeat the ethanol cleaning two more times.

27. After the final washing keep the tubes open (about 5 min) to let the remains of ethanol evaporate.

28. When the bead-pellet look like mud add 21 µl EB.

29. Vortex, pulse spin and incubate for 5 min.

30. Place the tubes on a magnetic rack/plate.

31. When all beads are attached to the wall move the supernatant to new tubes (it contains the ligated DNA) and discard the beads.

### *Alternative purification with MinElute for low concentration and poor samples (see above)*

## Library preparation step 3 - Adapter fill in(40 µl final Volume/reaction)

Prepare a master mix for the required number of ligation reactions as shown below. Keep enzymes on ice. Finally mix 20 µl of the master and 20 µl of the ligated DNA per reaction.

| Reagent | Volume/sample [µl] |  |  |
| --- | --- | --- | --- |
| ddH_2_O | 14.1 |  |  |
| Thermopol buffer (10X) | 4 |  |  |
| dNTPs (25 µM each) | 0.4 |  |  |
| *Bst* polymerase, LF (8 U/μL) | 1.5 |  |  |
| Total master mix: | 20 µl |  |  |

32. Mix and incubate for 20 min at 37 °C and heat kill for 20 min at 80 °C.

*This is the final library without indices. It can be kept in the freezer before Index PCR.*

## Index PCR

*Note: It is favorable to run as few cycles as possible during the index PCR, as increased number of cycles increase the clonality, and that running multiple independent PCR on each library is a way to increase the complexity.*

In order to assess how many PCR cycles are needed for each library it is optional to first prepare a 12 cycle PCR-reaction per library and then check it on a gel. If the band is too bright one could set up the final PCR reactions with a lower number of cycles (e.g., 8-10). If, on the other hand, the band is too weak or invisible, then the final PCR reactions can be set up with a higher number of cycles (e.g., 14). In the final PCRs, it is recommended to use unique dual primer combinations to ensure that potential index hopping could be detected.

33. In total set up 4-6 reactions per DNA library in 25 µl reactions containing:

| Reagent | Volume/sample [µl] |  |  |
| --- | --- | --- | --- |
| ddH_2_O | 16-18 (depending on library volume, e.g. 16 µl for 5 µl of DNA) |  |  |
| AccuPrime reaction mix (10X) | 2.5 |  |  |
| Index primer P5 (10µM) | 0.5 |  |  |
| Index primer P7 (10µM) | 0.5 |  |  |
| AccuPrime Pfx | 0.5 |  |  |
| Library DNA | 3-5 (higher volumes may reduce the number of PCR-cycles) |  |  |
| Total volume: | 25 µl |  |  |

34. Mix and spin down and use the following thermal cycler program

| Step | Temperature [°C] | Time |  |
| --- | --- | --- | --- |
| Initial denaturation | 95 | 2 min |  |
| Denaturation | 95 | 15 s |  |
| Annealing | 60 | 30 s | 12 cycles* |
| Elongation | 68 | 60 s |  |
| Hold | 4 | ∞ |  |

**Denotes recommended number of cycles for the library PCR test*

# Final AMPure XP purification

*AMPure beads are used to for size selection of DNA. The following protocol was developed for Meyer-Kircher libraries on fragmented DNA (museum samples or ancient DNA). Modifications of the protocol below could be used to make size selection/and or to concentrate DNA-extracts or PCR-products etc. Different fragment size cut offs can be achieved by adjusting the AMPure beads:DNA ratio (see manufactory protocols for details).*

## Preparations

- Take the AMPure bead bottle out of the fridge and let it come to room temperature. This takes about 30 min.
- Pool together all PCR reactions of each library in a clean low-binding Eppendorf tube. Allocate the volume to be purified (e.g. 100 µl) of the PCR product to another low-binding tube.
- Prepare fresh 70% EtOH and vortex it thoroughly. It must be fresh to make sure the EtOH concentration is correct.
- Prepare EB buffer supplemented with 0.05% Tween-20 for the elution steps. (optional)

## Short fragment removal (Left side selection)

*Note: This step removes fragments around < 100 bp*

1. Thoroughly vortex the AMPure bead bottle and add 1:1 volume to the supernatant from the steps above or to the pooled PCR product.
2. Vortex, pulse spin and incubate in an ordinary rack at room temperature for 10 min. *Avoid pelleting (if pelleting, vortex and pulse spin again)*
3. Place the tubes on the magnetic rack and incubate for about 3 min.

*The migration is visible to the naked eye, so one can incubate for a longer or shorter time according to the state of the tubes*

1. Discard the supernatant and keep the pellet.

*The pellet now contains the library, and the supernatant contains smaller fragments such as additional primers, dNTPs, etc.*

1. Add 200 µl 70% EtOH to wash the pellet.
2. Vortex briefly, pulse spin and place the tube rack on the magnetic rack.

*EtOH tightens the bond between the AMPure beads and DNA by changing the electrostatic charge of the magnetic beads, so it is fine if the pellet remains undissolved during this step*

1. Discard the supernatant and repeat steps 5-6 two more times.

*Three washing steps in total, make sure you remove all ethanol droplets after the third washing step*

1. Open the lids of the tubes and let the EtOH vaporize completely from the pellet.

*The bead pellet should look like mud and if it is shining, there is still EtOH remaining. This could take 5-10 minutes. If the pellet gets too dry, the yield will decrease.*

1. Elute DNA from the pellets in 50 µl EB Buffer (preferably supplemented with 0.05% Tween-20).

*EB buffer (or water) also changes the electrostatic charge of the beads, resulting in a loosening of the bond between beads and DNA*

1. Vortex for 20 s, pulse spin and incubate in an ordinary rack at room temperature for 5 min.

*Make sure the beads are released before pulse spinning*

1. Put the elution on the magnetic rack and incubate for 5 min.

*This step will separate empty beads from the DNA*

1. Transfer the supernatant to a clean low-binding Eppendorf tube.
2. Run 1 µl of the cleaned library on a Qubit (aim for 5 ng/ µl and dilute accordingly)
3. Run another 1 µl of cleaned (if necessary diluted) library on a Bioanalyzer for quantification.
